# Short-term adjunctive use of a liposomal tear substitute after repeated intravitreal therapy: a randomized pilot study

**DOI:** 10.1007/s10792-026-04249-7

**Published:** 2026-09-16

**Authors:** Carlo Cagini, Marco Messina, Tito Fiore, Maria Luisa Chiruzzi, Martina Frenguelli, Luigi Crescenzi, Maria Poddi

**Affiliations:** Department of Medicine and Surgery, Ophthalmology Section, S. Maria Della Misericordia Hospital, University of Perugia, Perugia, Italy

**Keywords:** Ocular surface, Tears, Macula, Macular degeneration, Cornea, Conjunctiva

## Abstract

**Purpose:**

This pilot study investigates the efficacy of a liposomal ophthalmic solution containing cross-linked hyaluronic acid, trehalose, and stearylamine in reducing the occurrence of dry eye-like signs and symptoms in patients undergoing repeated intravitreal treatment.

**Methods:**

Participants aged over 50 years old were randomly assigned to receive either standard therapy alone (Group 1) or standard therapy with additional eye drops (Group 2). Examinations included TBUT, Schirmer’s test, fluorescein staining, the Ocular Surface Disease Index (OSDI), and the 5-item Dry Eye Questionnaire (DEQ-5) at baseline and day 15, with a 30-day telephone follow-up for OSDI reassessment.

**Results:**

Ninety patients with age-related macular degeneration or other retinal diseases requiring anti-VEGF (vascular endothelial growth factor) treatment were enrolled in the study. In Group 1, which comprised 20 males and 25 females, DEQ-5 scores did not exhibit any significant change between V0 and V1 (10.89 ± 2.71 to 10.57 ± 2.60, p = 0.173), while Group 2, composed of 18 males and 27 females, demonstrated a considerable reduction in DEQ-5 values (9.28 ± 3.24 to 8.00 ± 3.64, p < 0.0001). OSDI scores in Group 1 showed no relevant change between V0 and V1 (p = 0.497), whereas in Group 2 they improved significantly (p < 0.0001). Additionally, Group 2 had notable enhancements in TBUT and Schirmer tests (p < 0.0001), while Group 1 experienced a significant decrease in TBUT values alone (p = 0.026).

**Conclusion:**

Chronic intravitreal therapy contributes to significant ocular surface impairment, which can be effectively mitigated by the administration of a tear substitute alongside the standard treatment.

## Introduction

Intravitreal injections of anti-VEGF have become the standard of care for various macular and posterior segment pathologies [1]. For many of these conditions, intravitreal injections are administered on a chronic basis. As documented, this can lead to the development of dry eye disease (DED) in healthy patients or exacerbate a pre-existing dry eye condition [1, 2]. Indeed, intravitreal treatment is generally preceded by thorough disinfection of the corneo-conjunctival surface, and the subsequent potential toxic effects of this procedure are already well-known [3]. In addition, a role in the onset of ocular surface distress is also related to the use of medications that are prescribed pre- and postoperatively [3].

Recently, it has been described that patients undergoing intravitreal treatment for the first time tend to develop typical dry eye disease symptoms in the postoperative period. In this cohort, the use of tear substitutes effectively reduces post-treatment ocular discomfort [4]. The onset of symptoms related to dry eye, or the worsening of a pre-existing condition, is often overlooked in clinical practice. However, it is well established that these issues can significantly impact patients’ quality of life, especially for those receiving multiple intravitreal treatments throughout the year [5].

In this report, we evaluated the association and severity of ocular discomfort signs and symptoms in patients undergoing multiple cycles of intravitreal injections. Additionally, we assessed the effects of a liposomal ophthalmic solution containing cross-linked hyaluronic acid, trehalose, and stearyl amine on dry eye–like signs and symptoms as an adjunct to standard therapy. These eye drops have properties that make them particularly suitable for this purpose: sodium hyaluronate retains water and moisturizes the ocular surface; trehalose has a mild anti-inflammatory effect and a protective effect on cells by helping to restore osmotic balance on the ocular surface and reducing conjunctival inflammatory cytokines; and stearylamine increases the contact time and efficacy of the active ingredients.

## Materials and methods

This pilot randomized, parallel-group pilot study included patients requiring anti-VEGF treatment at the Ophthalmology Section of the Department of Medicine and Surgery of the University of Perugia. The study was approved by the competent Ethics Committee (n. 4614/23). Patients were invited to participate in the study if they were found to satisfy the inclusion and exclusion criteria. Patients over 50 years of age, diagnosed with exudative AMD, post-operative macular oedema and sequelae of retinal vein occlusion, who had undergone at least three intravitreal injections in the previous six months, were considered eligible for inclusion. Patients who were deemed non-compliant, were under systemic corticosteroids or non-steroidal anti-inflammatory drugs, with neovascular glaucoma, had certified contraindications to the use of the study drug, or had known allergies to the study drugs or their components, were not considered eligible for the study. Patients with topical eye drop use within three months prior to the study or chronic topical therapy for any purpose were also excluded, as well as those with ocular allergy, blepharitis/MGD, autoimmune disease, Sjögren's syndrome, ocular surgery within 12 months prior to the study, punctal plugs, topical cyclosporine/lifitegrast/steroids, or contact lens wear.

Female patients who were pregnant, breastfeeding, or planning to become pregnant, as well as those who had received another investigational drug or device within 30 days of enrollment, were also excluded.

A written informed consent was obtained from all patients during their enrollment in this study. All patients underwent an ophthalmological examination at the baseline visit (V0), including best corrected visual acuity, intraocular pressure, slit-lamp biomicroscopic examination, fundus examination, and retinal tomography imaging. Additionally, the tear break-up time (TBUT) test, Schirmer's I test, and fluorescein staining test were performed at the baseline visit (V0), and the DEQ-5 and OSDI questionnaires were administered as well. The decision to use both the DEQ-5 and OSDI tests in our practice is due to their differing sensitivity in cases of mild dry eye and the influence that visual function may have on the score obtained. The TBUT test and the Schirmer I test, which were performed to assess tear film stability, tear film quality, and total tear secretion, were conducted as detailed below: TBUT was assessed after instillation of 5 μL of 2% sodium fluorescein using a slit-lamp biomicroscope equipped with a cobalt blue filter. The time interval between the last complete blink and the first appearance of a dry spot on the cornea was recorded in seconds, and the mean of three measurements was considered. The Schirmer I test was performed using standard sterile strips (Whatman filter paper No. 41) placed in the lateral third of the lower fornix for 5 min; the wetted length was measured in millimeters. Corneal and conjunctival fluorescein staining was graded using the Oxford scale (0–5). The Ocular Surface Disease Index (OSDI) and Dry Eye Questionnaire−5 (DEQ-5) were administered in their validated Italian versions.

At the baseline visit (V0), all patients were allocated to one of the study groups using a systematic quasi-randomization method based on an 'odd or even' rule, where odd numbers (1, 3, 5) were assigned to Group A, and even numbers (2, 4, 6) to Group B.

Subsequently, patients underwent the intravitreal injection. After the application of an adhesive sterile drape, a blepharostat was applied and 3 to 4 drops of topical lidocaine 40 mg/ml (Lidocaine Hydrochloride Intes—Alfa INTES Industria Terapeutica Splendore, Italy) were instilled, followed by the application of povidone-iodine 50 mg/ml (Oftasteril—Alfa INTES Industria Terapeutica Splendore, Italy), which was left in place for 3 min. After the intravitreal injections, patients were instructed to begin postoperative therapy. For Group 1, this consisted of standard postoperative therapy (Iodim sterile eye drops—10 mL MEDIVIS Srl, Italy), administered 3 times a day for 3 days. Group 2 received the same standard postoperative therapy for 3 days plus the study eye drops — a tear substitute formulation containing cross-linked hyaluronic acid, trehalose, and stearylamine in a liposomal ophthalmic solution buffered to physiological pH (Trimix Eye Drops, Offhealth S.p.a., Italy) — instilled 3 times a day for 10 days.

Following intravitreal treatment, patients were monitored according to the standard protocols established for all patients undergoing this procedure. In addition, study participants underwent specific testing to identify any potential alterations. The day after treatment, all patients underwent a routine follow-up visit. A second follow-up visit (V1) was scheduled 15 days after the intravitreal injection, during which both objective (TBUT test, Schirmer's test, fluorescein staining test) and subjective tests (DEQ-5, OSDI questionnaires) were performed. Finally, patients underwent a telephone interview (V2) 30 days after the intravitreal injections, in which the OSDI questionnaire was administered.

The primary endpoint of the study was to evaluate the change in DEQ-5 score between visits V0 and V1. Secondary endpoints included assessing changes in OSDI scores across visits V0, V1, and V2; measuring alterations in TBUT, Schirmer's I test results, and fluorescein staining between V0 and V1; and recording the occurrence and severity of any adverse events throughout the study.

Patient randomization and treatment allocation were performed by personnel who were not involved in patient recruitment, treatment, or clinical evaluation. Furthermore, the investigators evaluating the patients were blinded to the study group assignments. The examiners were masked at the baseline visit and during all subsequent follow-up assessments. All evaluations were conducted in strict accordance with the guidelines established by the Tear Film & Ocular Surface Society (TFOS) and reported in the DEWS and DEWS II reports.

### Statistical analysis

Quantitative variables were described by their mean and standard deviation (SD). The Shapiro–Wilk test was used to check whether the sample was drawn from a normally distributed population. Depending on the statistical distribution, comparisons of means of independent variables were performed using either the Student’s t-test or the Mann–Whitney test. Longitudinal analysis was conducted using the paired t-test or the Wilcoxon paired test. Data related to the OSDI questionnaire were analyzed using non-parametric tests (Wilcoxon and Friedman tests).

In the comparative analysis between the groups, data are presented as the mean of differences and standard deviation (S.D.), the mean difference between groups, and the 95% confidence interval. Given the distribution characteristics of certain variables, statistical comparison between Group 1 and Group 2 was conservatively performed using the non-parametric Mann–Whitney U test. The effect size was estimated using Cohen’s (r =|Z|/√N), with the effect magnitude classified as small ($r\ge \mathrm{0,1}$), medium ($r\ge \mathrm{0,3}$), or large ($r\ge \mathrm{0,5}$).

A p-value of <0.05 was considered statistically significant. All statistical analyses were performed using the SAS System for Windows (release 9.4, SAS Institute Inc., Cary, N.C., USA).

### Sample size

The sample size was calculated based on the study's primary objective, assuming a minimum difference of 2 points on the DEQ5 questionnaire between the groups (untreated and treated), a standard deviation of 2.50 and 3.60, respectively, a significance level (α) of 5%, a statistical power of 80%, and a 1:1 sampling ratio between groups. The estimated minimum sample size was 40 subjects per group.

## Results

In this study, 90 patients were enrolled and divided into two groups. Group 1 included 45 patients (20 males and 25 females, mean age 77 ± 8) who underwent an average of 12.1 ± 9.1 intravitreal treatments and received standard postoperative therapy alone. Group 2 included 45 patients (18 males and 27 females, mean age 76 ± 11) who went through an average of 13.0 ± 10.6 intravitreal injections and received a tear substitute in addition to standard postoperative treatment. After treatment, no patients reported any significant lesions at the injection site, infections, or any adverse reactions, either in relation to the standard therapy or the eye drops under study.

An intragroup comparison between baseline (V0) and post-treatment (V1) values was performed. Group 1 showed no relevant change in any of the parameters studied between V0 and V1; we did not observe any statistically significant changes in DEQ-5, OSDI, TBUT, Schirmer test, or fluorescein staining values between the two measurements performed (Tables 1, 2, 3, 4, 5, 6).

**Table 1:** DEQ5 questionnaire values at V0 and V1 in group 1 and 2

	V0	V1	p value
Group 1	10.89 ± 2.71	10.57 ± 2.60	0.173
Group 2	9.28 ± 3.24	8.00 ± 3.64	< 0.0001

**Table 2:** OSDI questionnaire values at V0 and V1 in group 1 and 2

	V0	V1	p value
Group 1	36.71 ± 2,67	32.63 ± 2.82	0.155
Group 2	43.57 ± 3.39	42.51 ± 3.18	< 0.0001

**Table 3:** OSDI questionnaire values at V0 and V1 in group 1 and 2

	V0	V1	p value
Group 1	36.71 ± 2,67	32.26 ± 2.79	0.272
Group 2	43.57 ± 3.39	42.18 ± 3.17	< 0.0001

**Table 4:** TBUT values at V0 and V1 in group 1 and 2

	V0	V1	p value
Group 1	6.02 ± 3.41	5.30 ± 2.70	0.026
Group 2	6.41 ± 5.14	7.28 ± 4.55	< 0.0001

**Table 5:** Schirmer test values at V0 and V1 in group 1and 2

	V0	V1	p value
Group 1	9.34 ± 6.61	8.48 ± 5.61	0.237
Group 2	10.10 ± 6.52	11.65 ± 6.99	< 0.0001

**Table 6:** Fluorescein staining values at V0 and V1 in group 1 and 2

	V0	V1	p value
Group 1	2.23 ± 0.91	2.27 ± 0.87	0.420
Group 2	2.04 ± 0.90	2.02 ± 0.86	0.569

Group 2 demonstrated a statistically significant decrease in DEQ-5 scores (p < 0.0001) (Table 1) and a statistically significant improvement in TBUT and Schirmer test values between V0 and V1 (p < 0.0001) (Tables 4, 5). Moreover, the OSDI parameters exhibited a statistically significant decrease both between V0 and V1, and between V0 and V2 (Tables 2, 3).

A between group values comparisons was performed: as shown in Table 7, the most pronounced and clinically relevant differences between the two groups occur in the tear break-up time (TBUT) and Schirmer test results. Specifically, TBUT not only reveals a highly significant difference between the groups (p < 0.0001) but also yields a large effect size, indicating minimal overlap and clearly distinct clinical profiles. A similar trend is observed for the Schirmer test. Regarding the DEQ-5 and OSDI, both variables demonstrate statistically significant differences, albeit with a moderate effect size (r). Lastly, the staining test results show no statistically significant differences, accompanied by a small effect size.

**Table 7:** Between group values comparisons

	Mean change Group 1	Mean change Group 2	group difference in change Group 1–Group2	95% confidence intervals	p value*	Effect size (r)
DEQ5	− 1.28 ± 2.1	− 0.35 ± 1.5	− 0.96	− 1.73–0.19	0.0139	0.26
BUT	0.87 ± 1.3	− 0.73 ± 2.1	1.59	0.87–2.32	< 0.0001	0.47
SCHIRMER	1.59 ± 2.5	− 0.86 ± 4.8	2.45	0.86–4.04	0.0001	0.41
STAINING	0.02 ± 0.3	0.05 ± 0.4	− 0.67	− 0.2–0.07	0.3362	0.1
OSDI	− 3.6 ± 5.7	− 1.20 ± 6.00	− 2.46	− 4.9–0.01	0.0326	0.23

## Discussion

This randomized study showed that patients undergoing repeated intravitreal injections frequently experience dry eye–like symptoms. The postoperative use of eye drops containing cross-linked hyaluronic acid, trehalose, and stearylamine, which act as tear substitutes, leads to improvement in these symptoms. Anti-VEGF intravitreal injections are now a standard of care for many ophthalmic conditions regarding a variety of posterior segment pathologies and are mostly administered on a chronic basis [1, 2, 6]. Given the repetitive nature of intravitreal anti-VEGF treatment and the age-related susceptibility to dry eye disease (DED), awareness of the long-term effects of intravitreal injections on the ocular surface is advisable [6].

Indeed, many studies have outlined the tendency of surgical procedures to induce the onset or the worsening of dry eye disease [1, 2]. In this regard, a consensus has been reached about the proneness to dry eye disease after cataract surgery, which has been shown to induce an upregulation of inflammatory mediators in the postoperative period, altering the sensitivity of the ocular surface nerve plexuses and leading to a persistent loss of conjunctival goblet cells [7–9]. The correlation between surgical procedures and the subsequent development of ocular surface impairment has been attributed, on one hand, to the pre-injection antisepsis with povidone-iodine performed to minimize the risk of endophthalmitis, which conversely damages the corneal epithelium and Meibomian glands [2, 10, 11]. As previously demonstrated, these harmful effects are also related to the perioperative use of anesthetic and antiseptic eye drops [7, 12].

There is also evidence that patients undergoing intravitreal treatment for the first time, with a healthy ocular surface and treated with a tear substitute in addition to standard postoperative therapy, experienced a significant improvement in DEQ-5 and OSDI questionnaire results, leading to important relief from subjective discomfort [4]. However, the brief exposure to ocular surface toxic agents was not sufficient to induce an impairment that would alter objective tests such as TBUT, Schirmer, and fluorescein staining tests [4].

The distinctive aspect of this study is that it involved participants with varying degrees of dry eye disease. The analysis of the subjective parameters assessed by the two questionnaires revealed an improvement in the group treated with the artificial tear under investigation. Group 2 achieved such an improvement in subjective discomfort that patients shifted from having a moderate disease to a mild disease according to DEQ-5 scores, and from severe to moderate disease concerning OSDI scores [13]. "Based on our findings, the use of a tear substitute can provide clinical benefits not only regarding perceived dry eye symptoms but also for objective tests commonly used in clinical practice.

Regarding clinical-objective parameters (TBUT, Schirmer, and staining), the two groups exhibited opposing trends in numerical values. The study group (Group 2) showed significant improvements in TBUT and Schirmer scores (p < 0.0001), except for the fluorescein staining test (p = 0.57); this may be attributable to the enrollment of patients whose ocular surfaces are subjected to continuous toxic insults, suggesting that the use of artificial tears for the limited postoperative period was not sufficient to improve this parameter as well. On the other hand, in the control group (Group 1), TBUT, Schirmer, and fluorescein staining values decreased, with statistical significance noted solely for TBUT. Therefore, given that most surgical procedures are involved in the exacerbation or worsening of dry eye disease by altering the delicate tear film homeostasis, it would be advisable to use an artificial tear with specific features to achieve overall relief for the ocular surface.

This investigation highlighted the benefits of a tear substitute with a combination of viscosity-enhancing hyaluronic acid, trehalose, and cationic liposomes comprising stearylamine and phospholipids in patients undergoing multiple intravitreal treatments. Specifically, these lubricant eye drops belong to the group of multiple-action tear substitutes [13, 14]. In recent years, there has been a shift in the treatment and management of dry eye disease from simple water-adding medications to multi-action combined formulas, which are currently emerging as the therapy of choice by targeting different pathophysiological mechanisms [15, 16].

The artificial tear administered to patients enrolled in this study is specifically formulated to restore and maintain a structurally and functionally healthy ocular surface, owing to its unique components [17]. Liposomes deliver both non-polar and polar lipids to restore and thicken the lipid layer, enhancing stability at the interface with the aqueous phase of the tear film. Cross-linked hyaluronic acid provides mechanical protection, hydrates, and ensures long-lasting surface lubrication, while trehalose helps preserve cellular proteins from dehydration and guards against oxidative damage. Additionally, trehalose accelerates the healing process by reducing inflammatory cytokines and restoring the osmotic balance of the ocular surface [18–21]. The positive charge from stearylamine generates electrostatic forces that facilitate the uniform diffusion of the tear substitute over the negatively charged ocular surface [22].

However, this preliminary study has several limitations that should be acknowledged. The relatively small sample size (90 patients) and the short follow-up period of 15 days were chosen to evaluate the immediate safety and efficacy of the intervention in a heterogeneous patient population already undergoing treatment. Given these constraints, stratification based on clinical or demographic variables was not feasible, as subdividing the cohort into multiple subgroups would have resulted in very small sample sizes per group, compromising the statistical power of subgroup analyses. Moreover, the primary aim of this initial investigation was to assess overall effectiveness and safety rather than to analyze differential responses within specific patient subpopulations, which would require larger, more structured studies. We recognize that studies with larger populations, longer follow-up, and stratified analyses are important and plan to incorporate these in future, adequately powered research to better understand long-term effects and subgroup-specific responses. Another limitation is the absence of a control eye drop for Group 1—as this would have required a much larger number of patients stratified according to the severity of their preoperative condition—and the absence of an osmolarity study of the tear film, which could enhance our understanding of the observed results. These factors underscore the necessity for further research to validate our findings and deepen our insight into the outcomes achieved.

To our knowledge, this is the first comprehensive analysis of the changing trends in both objective and subjective parameters of dry eye-like symptoms in patients receiving repeated anti-VEGF intravitreal therapy, and our findings highlight the beneficial effects of using an artificial tear solution when administered for a short period in the postoperative period. Although these findings need to be confirmed by larger studies that include stratification of the patient population according to the severity of dry eye, our data suggest that the administration of an artificial tear can be considered for patients undergoing cycles of intravitreal treatment. Our study confirms, in fact, that following intravitreal treatments, the ocular surface may experience distress manifesting as dry eye-like symptoms, while also highlighting the importance of considering patient discomfort and the opportunity to administer a tear substitute.

## Data Availability

No datasets were generated or analysed during the current study.
